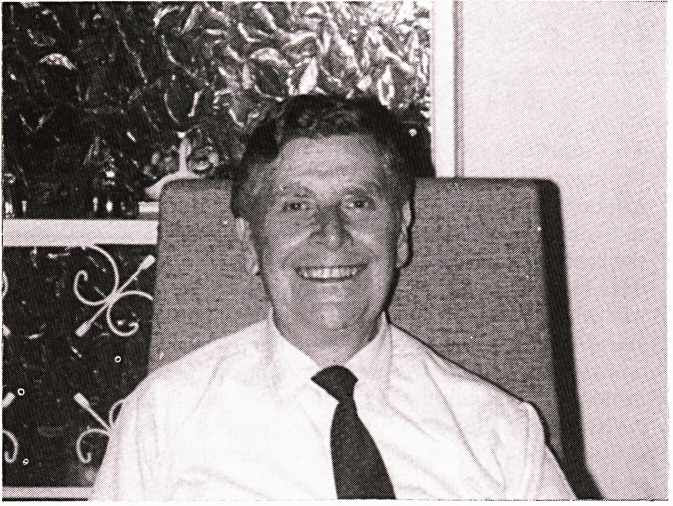# Dr Dennis Wright

**Published:** 1988-08

**Authors:** R. V. Walley


### OBITUARY

**Dr Dennis Walter Wright**

M.B. ChB (Bristol) 1952 A.F.O.M. RCP (Lond) 1978

Dr Dennis Wright, who died on 6th August 1988 in Ham Green hospital at the age of 61, played a large part in the work of the hospital and in the Avon branch of the British Red Cross Society. He qualified in 1952 in Bristol. As a resident he worked at Southmead Hospital in general medicine, in obstetrics under Professor Lennard, and in the casualty department, finishing as medical registrar to Dr Freddie Sutton and Dr Tony Birrell. Coming later to Bristol, I always enjoyed his stories of the foibles of his senior colleagues. Dennis had an enduring interest in his fellow men.

After working in the geriatric department at Manor Park, he came to Ham Green as assistant physician in 1959 when I first met him. In addition to his official work on the wards with Dr Bill Barritt and Dr Jimmie Macrae he made a large contribution to most other aspects of the life of the hospital. Living initially in a flat in the grounds with his wife Iris and their two daughters, he was in close touch with all that went on. I myself worked at Ham Green for many years, but it was a rare occasion when I was able to tell Dennis of any event he did not already know. With his wide experience of the hospital he was able to give help to most of us at some time or another: in particular he and Iris ran an excellent health service for all members of the staff which contributed much to the good atmosphere and smooth running of the hospital.

In addition to his work at Ham Green, Dennis, always a sociable man, had many interests and friends outside the hospital. This was apparent at his funeral when the church at Portishead was full to overflowing on a sunny spring day. Prominent among the mourners was an official party from the British Red Cross Society. Dennis had worked for forty-five years for the Society, starting before his medical training, becoming the Medical Officer for the Avon branch. In 1977 he received the Jubilee medal. I recall he particularly enjoyed working at the big occasions such as the Badminton Horse Trials when his wide medical and even his obstetric experience was put to good use. He was a regular correspondent to the Bristol Medico-Chirurgical Journal.

I have not mentioned his personal health problems because Dennis rarely mentioned them either, but they were severe and multiple. He had a courageous and cheerful spirit which was more than strong enough to put them in their place and allowed him to enjoy his life and work. In this he was helped by his wife and their daughters.

R. V. WALLEY

## Figures and Tables

**Figure f1:**